# Differences in Perceived Stress, Subjective Well-Being, and Psychosocial Variables by Game Use Type

**DOI:** 10.3390/bs14121178

**Published:** 2024-12-10

**Authors:** Goo-Churl Jeong, Kwanhyeong Kim, Bee Kim

**Affiliations:** 1 Department of Counseling Psychology, College of Creativity and Convergence, Sahmyook University, Seoul 01795, Republic of Korea; gcjeong@syu.ac.kr; 2 Department of Counseling Psychology, Graduate School, Sahmyook University, Seoul 01795, Republic of Korea; skctjsgksmf@syuin.ac.kr; 3 Department of Addiction Science, Graduate School, Sahmyook University, Seoul 01795, Republic of Korea

**Keywords:** game, game use, stress, well-being, family, parenting

## Abstract

This study examined the differences in perceived stress, subjective well-being, psychosocial variables, and differences in parents’ parenting styles according to game use type among Korean adults. The study involved 300 participants in their 20s and 30s, a demographic typically associated with frequent gaming. Data were collected through an online survey company, and analyses were conducted using SPSS 25.0, including correlation, cluster, ANOVA, and correspondence analyses. The results showed that the general and adaptive game use groups had significantly lower levels of perceived stress than the maladaptive and risky game use groups. Additionally, the adaptive game use group exhibited significantly higher subjective well-being than the maladaptive game use group. In terms of psychosocial characteristics, except for the general game use group, none of the other groups considered online gaming as addictive. Parenting styles showed significant differences in relation to game use in adulthood. Notably, democratic parenting styles were associated with the general and adaptive game use groups, whereas neglectful parenting styles were linked to the risky game use group. These findings suggest that the risky game use group is as vulnerable to stress as the maladaptive game use group, emphasizing the need for targeted screening and social attention for the risky game use group.

## 1. Introduction

Today, gaming has become a significant leisure activity for adults in their 20s and 30s. In 2023, more than half of Korean adults regularly engaged in gaming, with a growing trend among adults in their 20s and 30s to spend their leisure time playing various games [1]. For adults in this age group, the digital environment is a familiar space, and gaming provides opportunities to relieve everyday stress and burden. Notably, 81.7% of the parents of school-aged children reported playing games with their children [2]. This indicates a shift in the perception of gaming, previously considered as “child’s play” or viewed negatively as a “disease” or “pathological issue”. In modern society, gaming has expanded its role, serving not only as a personal leisure activity, but also as a platform for economic and social activities. In the 2023 Hangzhou Asian Games, E-sports was introduced as an official medal event, with the public showing their support for the competitors striving to win the gold medal [3]. This highlights the rapid evolution of perspectives on gaming over the past few decades.

### 1.1. Diverse Perspectives on Gaming

Traditionally, research on gaming has predominantly focused on pathological issues. In particular, studies on gaming addiction and its classification as a disorder have been highly active in Korea. Meta-analytic findings indicate that research on gaming addiction and maladaptive game use has been significantly more prominent in Korea compared to other countries [4]. Early studies primarily focused on psychological and emotional states such as depression and anxiety as well as problem behaviors such as aggression, addiction, and delinquency among game users [1,4]. Additionally, maladaptive game use has been associated with a lack of self-control, maladjustment, and the deterioration of social relationships [5,6]. Recent studies have raised concerns about the severity of gaming addiction, reporting that it can impair cognitive function and emotional regulation [7].

However, research focusing on the positive aspects of gaming has begun to emerge. According to studies on adaptive game use, gaming has the potential to enhance users’ problem-solving abilities, social interaction skills, and psychological stability [8]. From a positive psychological perspective, gaming can help reduce stress and foster social interaction [9]. These studies suggest that gaming can improve users’ self-efficacy and social trust [10], with an increasing number of reports indicating that individuals can enjoy gaming without overindulgence, leading to healthy lives [11]. These findings imply that the impact of gaming on life varies depending on individual characteristics and psychosocial factors and the effects of gaming differ according to game use type.

### 1.2. Relationship Between Adaptive and Maladaptive Game Use

Adaptive and maladaptive game use are opposing concepts. The perspective that classifies gaming addiction as a disease frames maladaptive game use as a disorder requiring treatment. The World Health Organization (WHO) has included “game disorder” in the International Classification of Diseases (ICD-11) [12], providing a basis for the development of prevention and treatment programs for gaming addiction. Previous studies have documented positive case studies in which patients overcame gaming addiction through treatment without transitioning to other maladaptive game use, such as smoking or pornography [13]. However, the perspective of viewing game use as a disease has faced criticism for overmedicalization, arguing that it is difficult to distinguish between game immersion and pathological addiction owing to ambiguous criteria [14].

Research on adaptive game use has reported cases in which gaming serves as a tool for stress relief and emotional regulation, helping users recharge their lives [2]. If adaptive and maladaptive game use are truly opposing concepts, research should show a negative correlation between them. However, the actual results contradict this expectation. Studies on college and graduate students have shown a significant positive correlation between adaptive and maladaptive game use (r = 0.55) [15], while similar results have been reported in studies on adolescents (r = 0.40) [16]. These findings suggest that higher maladaptive game use is associated with higher adaptive use, which appears counterintuitive. To address this issue, researchers have identified groups exhibiting both adaptive and maladaptive game use, with the resulting group perceived more positively than the maladaptive game use group [1]. However, we continue to encounter individuals who, while engaging in maladaptive game use, justify their behavior by claiming that it helps relieve stress and serves as an emotional escape. This dual behavior poses a significant risk; it makes it difficult to identify those who need intervention, and the individuals themselves may not recognize their behavior as problematic. Therefore, it is important to determine whether adaptive game use can offset maladaptive tendencies.

Approaches to understanding adaptive and maladaptive game use through psychological theories have been steadily developed. The approach based on the Flow Theory posits that game players can achieve a positive state of immersion through “optimal experiences,” indicating that games, which provide a strong sense of engagement, allow users to experience high levels of concentration and satisfaction, leading to positive outcomes [17]. However, when the state of immersion shifts into pathological addiction, it can result in escapism and functional impairment in daily life. Kim and Boo found that while immersion in gaming can offer positive experiences, it can also manifest symptoms of pathological addiction [18]. This suggests that immersion is not inherently positive and can vary depending on an individual’s psychological traits and the purpose of gaming use. The approach rooted in the Self-Determination Theory emphasizes autonomy and intrinsic motivation as core elements of adaptive game use. When gaming satisfies a user’s intrinsic needs and leads to autonomous motivation, it results in positive and developmental experiences [19]. Conversely, if these needs are unmet and gaming is driven by external motivation, it can lead to maladaptive game use [20,21]. This indicates that game use is influenced not simply by the time spent but by psychosocial factors and motivational differences.

### 1.3. Game Use and Classification of Game Use Types

Existing studies on classifying game use types have sought to explore the unique characteristics of each type by combining various aspects of game use tendencies, rather than simply dichotomizing game use into adaptive and maladaptive. For instance, Kim and Boo [18] categorized online game user groups into four types based on the average levels of game immersion and addiction: “high immersion-low addiction”, “high immersion-high addiction”, “low immersion-low addiction”, and “low immersion-high addiction”. Additionally, other studies have employed comprehensive diagnostic tools for game use to segment game user groups into “general game use group”, “adaptive game use group”, “maladaptive game use group”, and “at-risk maladaptive game use group”. The general game use group is characterized by low levels of adaptive and maladaptive game use, while the adaptive game use group exhibits high levels of beneficial use without associated issues. In contrast, the maladaptive game use group has low levels of adaptive game use but high levels of maladaptive game use, and the at-risk maladaptive game use group shows high levels of both adaptive and maladaptive game use [1]. Seo and Lee [22] classified game user groups based on game immersion and addiction into “high immersion group”, “coexisting immersion-addiction group”, and “low immersion group”, and investigated psychological traits and the influence of autonomy within these groups. These multi-dimensional classifications have contributed significantly to an in-depth understanding and analysis of the complexity of game use.

Previous studies have attempted to consider both the positive and negative aspects when classifying game users, but they have often focused primarily on the duality of immersion and addiction. However, it is important not to overlook that game use often occurs at a normal level that does not lead to excessive immersion or addiction [22]. Therefore, it is necessary to move beyond merely distinguishing between game users and non-users and to compare classifications based on levels of immersion and addiction with general game use to broaden the understanding of game users. Thus, this study aims to classify game use types in detail based on adaptive and maladaptive game use. While some studies have used predefined cutoff scores for classification [1,16], adaptive and maladaptive game use do not always manifest to the same extent. It is essential to classify groups based on their specific characteristics. To achieve this, we conducted a cluster analysis using independently developed scales for adaptive and maladaptive game use. This classification provides a clearer understanding of game use characteristics among Korean adults.

### 1.4. Differences in Stress, Well-Being, and Psychosocial Variables by Game Use Types

Adaptive game use allows individuals to experience a sense of achievement, stress relief, and social connectedness, all of which can enhance their quality of life [23]. People who engage in adaptive game use often form new relationships or strengthen existing ones through online gaming [24], which has been shown to increase personal happiness and subjective well-being [25,26]. In contrast, maladaptive game use is characterized by loss of control over gaming, neglect of real-world responsibilities, and excessive dependence on gaming [1]. Those who engage in maladaptive game use often experience negative impacts on their self-control owing to real-life stress, leading to a decline in their quality of life and overall satisfaction [27]. Additionally, maladaptive gamers tend to experience weakened social relationships and a heightened sense of psychological isolation [28]. Individuals in the adaptive game use group exhibited lower levels of psychological factors such as aggression, depression, and loneliness compared to those in the maladaptive game use group. Conversely, they show higher levels of psychosocial factors such as self-control, self-esteem, social capital, and peer support [29]. Furthermore, parenting styles have been shown to negatively influence adolescent gaming addictions [30,31]. However, few studies have examined the relationship between psychosocial factors and game use type, with most studies focusing on adolescents. Research on adults, with regard to the diverse psychosocial factors related to game use, remains insufficient. Given the expanding role of gaming and its influence on adult relationships, further exploration of adult game use, particularly in relation to various psychosocial variables, is necessary.

This study aims to classify game use types based on adaptive and maladaptive game use among adults in their 20s and 30s, and to comprehensively explore the relationships between these game use types and psychological and social characteristics. First, this study hypothesizes that there will be correlations between adaptive game use, maladaptive game use, perceived stress, and subjective well-being. Second, it is expected that there will be differences in perceived stress and subjective well-being according to game use types. Lastly, the study aims to examine whether there are differences in psychosocial characteristics based on game use types.

The hypotheses of this study are as follows:

**Hypothesis** **1.** *There will be correlations between adaptive game use, maladaptive game use, perceived stress, and subjective well-being.*

**Hypothesis** **2.** *There will be a classification of game use types based on variables related to adaptive and maladaptive game use.*

**Hypothesis** **3.** *There will be differences in perceived stress and subjective well-being according to game use types.*

**Hypothesis** **4.** *There will be differences in psychosocial characteristics according to game use types.*

## 2. Methods

### 2.1. Participants

The study participants comprised 300 adults in their 20s and 30s residing in South Korea. The average age of the participants was 29.7 years (SD = 5.8), with 52.7% male and 47.3% female. To allow for age-group comparisons, 150 participants were selected from each age group (20s and 30s). Regarding marital status, 25.0% were married and 73.3% were unmarried. The most commonly used gaming devices were mobile devices (50.3%), followed by PCs (43%), handheld gaming devices (3.7%), and gaming consoles (2.3%), indicating that most participants played games on mobile devices or PCs. In a survey on perceptions of online gaming addiction, 63% of the participants did not consider themselves as addicted. Only 31.8% expressed satisfaction with their current economic status, while 69.0% considered themselves physically healthy, and 69.7% considered themselves mentally healthy. Regarding self-control, 67.3% of the participants reported high self-control. Additionally, 33.0% of the participants reported experiencing recent feelings of depressed mood, while 50.3% reported experiencing anxiety. In terms of family-related variables, 86% indicated that they had good family relationships and 68.7% reported effective family communication.

### 2.2. Measures

#### 2.2.1. Adaptive Game Use

Adaptive game use was measured using the Adaptive Game Use Scale (AGUS-β) from the Comprehensive Scale for Assessing Game Behavior (CSG-β), developed by the Korea Creative Content Agency [1]. The AGUS-β consists of seven factors: enjoyment and vitality, life experience expansion, emotion and stress management, immersion experience, challenge and achievement, control enhancement, and social interaction. Each factor contained three items, resulting in twenty-one items. Responses were measured on a 5-point Likert-type scale, with higher scores indicating greater adaptive game use. In this study, the reliability (Cronbach’s α) of adaptive game use was 0.94.

#### 2.2.2. Maladaptive Game Use

Maladaptive game use was assessed using the Internet Gaming Use-Elicited Symptom Screen (IGUESS), a self-reported screening tool developed based on the DSM-5 diagnostic criteria for Internet gaming addiction [32]. The IGUESS is a unidimensional scale consisting of nine items measured on a 4-point scale, with higher scores indicating a greater tendency toward gaming addiction. The reliability (Cronbach’s α) of maladaptive game use in this study was 0.93.

#### 2.2.3. Perceived Stress

Perceived stress was measured using the Korean version of the Perceived Stress Scale developed by Cohen et al. (1983) [33,34]. The Perceived Stress Scale is a unidimensional scale consisting of 10 items measured on a 5-point Likert-type scale, with higher scores indicating higher levels of perceived stress. In this study, the reliability (Cronbach’s α) for perceived stress was 0.77.

#### 2.2.4. Subjective Well-Being

Kim et al. (2009) developed a ten-item scale specifically designed to capture individuals’ perceptions of their own well-being, focusing on the subfactors of life satisfaction and life expectancy [35,36]. Participants expressed their subjective evaluations on a 7-point Likert-type scale, where higher scores reflected greater levels of perceived well-being. This scale has been extensively used in diverse research settings to provide a comprehensive understanding of how individuals internally perceive and assess their satisfaction with life and their expectations for the future. In this study, the scale demonstrated strong internal consistency, with a Cronbach’s α value of 0.94.

#### 2.2.5. Psychosocial Variables

To analyze the differences in psychosocial characteristics based on game use type, the following survey items were included in the investigation. First, regarding psychological variables, participants were asked whether they recognized the concept of online game addiction. They were also asked to evaluate their ability to control their behavior and to rate the frequency of experiencing depressed and anxious mood over the past six months using a 4-point Likert-type scale. Additionally, participants were asked to assess their satisfaction with their current economic status and their perceptions of their physical and psychological health. Regarding social variables, questions about family relationships, family communication, and parents’ parenting styles were included. These variables were examined to explore the influence of social factors on game use. Family relationships and family communication were measured using single items on a 4-point Likert scale and dichotomized for use in cross-tabulation analysis. Parenting styles were categorized into four types: permissive parent, democratic parent, authoritarian parent, and uninvolved parent. Participants were provided with descriptions of each parenting style and asked to evaluate the style that applied to their experience.

### 2.3. Procedure

We obtained ethical approval for this study from the Institutional Review Board (IRB No. SYU 2023-08-002). Data collection was carried out in August 2023 through an online survey conducted by a professional survey company, employing a nationwide sample. The survey utilized an anonymous questionnaire that did not collect any personally identifiable information. At the beginning of the online survey, participants were provided with a detailed explanation of the study. Only those who voluntarily agreed to participate and signed the online consent form were permitted to continue with the survey. The study exclusively involved individuals with prior gaming experience. While individual survey completion times varied, the average time to complete the survey was less than 30 min. 

### 2.4. Statistical Analysis

The collected data were analyzed using IBM SPSS Statistics for Windows (version 25.0; IBM Corp., Armonk, NY, USA). We present Cronbach’s alpha coefficients for the scales and descriptive statistics for the study variables. Correlations between variables were analyzed using Pearson’s product–moment correlation, and differences between variables were examined using Analysis of Variance (ANOVA), followed by post hoc testing using the Scheffé method. Hierarchical cluster and k-means cluster analyses were conducted to classify the game use groups. Differences in psychosocial variables across clusters were analyzed using the chi-squared test. To provide a clearer understanding of the relationship between parenting styles and game use groups, a correspondence analysis was performed, and a bi-plot was presented.

## 3. Results

### 3.1. Descriptive Statistics and Correlation Coefficients of Study Variables

The descriptive statistics and correlation coefficients of the study variables are presented in Table 1. Based on the skewness (−0.44 to 1.40) and kurtosis (−0.09 to 1.50) values of the study variables, normality was assumed. The analysis of correlations among the study variables revealed a significant positive correlation between adaptive and maladaptive game use (r = 0.37, *p* < 0.001). Additionally, adaptive game use was significantly and weakly positively correlated with subjective well-being (r = 0.13, *p* < 0.05), whereas no significant correlation was found between maladaptive game use and subjective well-being. Moreover, adaptive game use showed no significant correlation with perceived stress, whereas maladaptive game use was significantly positively correlated with perceived stress (r = 0.30, *p* < 0.001). Finally, perceived stress was significantly negatively correlated with subjective well-being (r = −0.57, *p* < 0.001). These results suggest that it is inappropriate to study adaptive and maladaptive game use separately. Therefore, this study aimed to analyze game use types by considering both adaptive and maladaptive game use.

### 3.2. Cluster Analysis to Categorize Game Use

**Figure 1:**
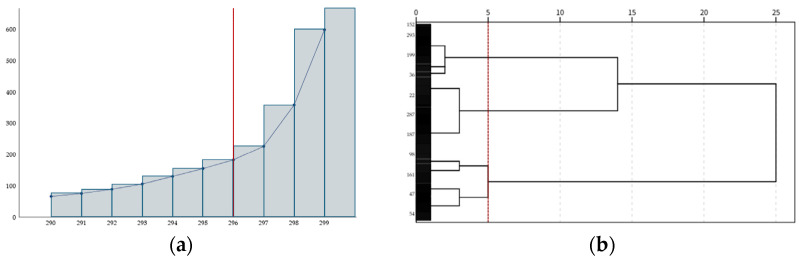
Hierarchical cluster analysis results: (**a**) Agglomeration schedule coefficients plot; (**b**) Dendrogram from hierarchical cluster analysis.

Game use types were classified using a cluster analysis based on adaptive and maladaptive game use scales. First, the number of clusters was identified through hierarchical cluster analysis, followed by the classification of groups using k-means cluster analysis. For hierarchical cluster analysis, adaptive and maladaptive game use variables were standardized and the Ward method was employed, utilizing the squared Euclidean distance. To determine the optimal number of clusters, the elbow method, which analyzes the changes in agglomeration coefficients, and a dendrogram were used. As shown in Figure 1a, the elbow point where a significant change in the agglomeration schedule coefficients occurred supported a four-cluster solution. In addition, the dendrogram (Figure 1b) indicated that the four clusters were appropriate. A previous study [1] also supported the classification of the four groups, and the formation of four clusters when using the two variables was predictable. Therefore, the final number of clusters was determined to be four.

**Figure 2:**
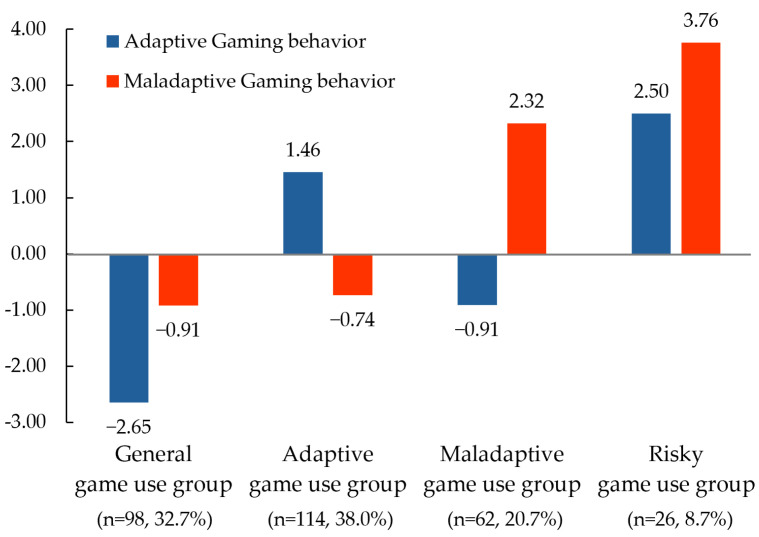
Final cluster centroid and game use type.

A k-means cluster analysis was performed using the four clusters identified through hierarchical clustering. Consequently, four cluster centroids were derived, as shown in Figure 2. Upon detailed examination, Cluster 1, characterized by both low adaptive and maladaptive game use, was labeled the “general game use group”, comprising 32.7% of the participants. Cluster 2, which displayed highly adaptive game use, was labeled the “adaptive game use group” (38.0%). Cluster 3, characterized by highly maladaptive game use, was labeled the “maladaptive game use group” (20.7%). Finally, Cluster 4, which exhibited both highly adaptive and maladaptive game use, was named the “risky game use group” (8.7%) based on terminology from previous studies [1].

Analysis of variance (ANOVA) was conducted to examine the differences in adaptive and maladaptive game use according to the game use types classified through cluster analysis. The results of the Scheffé post hoc test are presented in Table 2. Specifically, for adaptive game use, scores were significantly higher in the following order: general game use, maladaptive game use, adaptive game use, and risky game use (F(3.296) = 186.35, *p* < 0.001, η^2^ = 0.654). In contrast, for maladaptive game use, no significant differences were found between the general and adaptive game use groups, whereas the maladaptive and risky game use groups showed progressively higher scores (F(3.296) = 419.98, *p* < 0.001, η^2^ = 0.810). Notably, in the risky game use group, both adaptive and maladaptive game use scores were highest, indicating the importance of considering this group with caution.

### 3.3. Differences in Perceived Stress and Subjective Well-Being by Game Use Type

The results of the analysis of variance (ANOVA) for perceived stress and subjective well-being across game use types are presented in Table 3. Perceived stress was significantly higher in the maladaptive game use group and the risky game use group compared to the general game use group and the adaptive game use group (F(3.296) = 11.33, *p* < 0.001, η^2^ = 0.103). The adaptive game use group showed significantly higher levels of subjective well-being than the maladaptive game use group. However, for the risky game use group, post hoc tests indicated no statistically significant differences from the other groups (F(3.296) = 3.62, *p* = 0.014, η^2^ = 0.035). In summary, while the risky game use group perceived higher stress than the adaptive game use group, no significant differences in subjective well-being were observed.

### 3.4. Differences in Game Use Type by Demographic Variables

An analysis of gender differences across game use types revealed that the proportion of women in the general game use group was significantly higher at 47.9% compared to men, while the proportion of men was significantly higher in all other game use groups (χ^2^ = 30.13, *p* < 0.001). However, differences in age group, marital status, economic status satisfaction, and perceived physical and mental health across game use types were not statistically significant (Table 4).

### 3.5. Differences in Psychosocial Characteristics by Game Use Type

An exploration of the differences in game use according to psychosocial characteristics is presented in Table 5. Regarding the awareness of online game addiction, 62.2% of the general game use group recognized it as an addiction, whereas significantly higher proportions (66.1% to 80.8%) of the other groups did not recognize it as such (χ^2^ = 43.01, *p* < 0.001). The maladaptive game use group reported the highest proportion of low self-control at 48.4%, followed by the risky game use group at 38.5%, both of which were significantly higher compared to the general and adaptive game use groups (χ^2^ = 10.96, *p* = 0.012). The proportion of participants who experienced depressed mood was lowest in the adaptive game use group (24.6%), followed by the general game use group (31.6%). In contrast, the maladaptive game use group and risky game use group reported significantly higher rates of experiences of depressed mood at 45.2% and 46.2%, respectively (χ^2^ = 9.37, *p* = 0.019). Notably, the risky game use group, which scored high in both adaptive and maladaptive game use, exhibited higher depressed mood scores than the maladaptive game use group. There were no significant differences between groups in terms of anxious mood.

The percentage of participants reporting good family relationships was the highest in the adaptive game use group at 93.9%, followed by the maladaptive game use group at 82.3%, and the lowest in the risky game use group at 65.4% (χ^2^ = 15.89, *p* < 0.001). There were no significant differences in the degree of family communication between the game use groups.

### 3.6. Perceived Parenting Style and Game Use Type

The results of the chi-squared analysis between perceived parenting styles and game use types, presented in Table 6, indicate that four cells had expected counts of less than five. Therefore, the Fisher–Freeman–Halton Exact test was performed. The analysis yielded an exact *p*-value of 0.014, confirming a statistically significant difference between perceived parenting style and game use type. Upon closer examination of Table 6 for the democratic parenting style, the maladaptive game use group showed the lower proportion at 18.3%, while the risky game use group also exhibited a notably low percentage at 5.9%. In contrast, for permissive parenting style, the maladaptive game use group had the highest proportion (37.0%). Furthermore, the risky game use group had a particularly high proportion (33.3%) of neglectful parenting styles.

**Figure 3:**
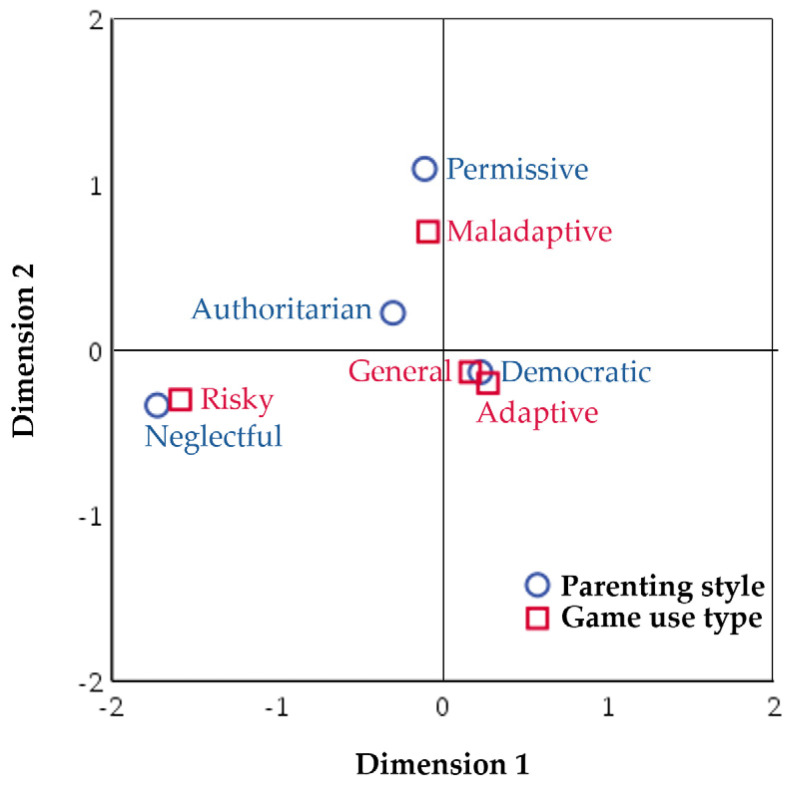
Biplot between perceived parenting style and game use type.

To provide a clearer understanding of the complex cross-tabulation between perceived parenting styles of parents and game use types, a correspondence analysis was conducted and visualized in a two-dimensional graph (Figure 3). The number of dimensions was set to two for simplicity, and given that the study variables were categorical, distances were measured using the chi-squared method with symmetric normalization applied. The results indicate that the proportion of inertia for the first dimension explains 77.9% of the total variance, whereas the second dimension explains an additional 21.2%, with the two dimensions accounting for 99.1% of the variance. This suggests that data patterns can be sufficiently explained by these two dimensions. In the biplot (Figure 3), the democratic parenting style was positioned very close to the general and adaptive game use groups, indicating a strong association. In contrast, neglectful parenting was closely aligned with risky game use. Additionally, both the authoritative and permissive parenting styles were located near the maladaptive game use group. These findings highlight the significance of democratic parenting in shaping adult game use, particularly emphasizing its positive influence in fostering adaptive game use.

## 4. Discussion

We analyzed differences in perceived stress, subjective well-being, and psychosocial characteristics according to game use type among Korean adults. The findings revealed significant differences in the perception of stress based on game use type among Korean adults in their 20s and 30s, with the adaptive game use group showing higher levels of subjective well-being. Additionally, there were notable differences in individual psychological and social variables across the game use types. Based on these results, the following conclusions are drawn.

The results of this study reveal that adaptive and maladaptive game use can coexist, indicating the necessity of classifying game use types using both variables rather than simplifying them into a dichotomy of positive and problematic use. Through a data-driven cluster analysis, four distinct groups were identified, which align with the classifications reported in prior studies [1,18]. Notably, earlier research that employed predefined cutoff points for group classification often viewed the risky game use group (characterized by high levels of both adaptive and maladaptive game use) as less problematic than the purely maladaptive game use group [1,16,37]. However, the data-driven cluster analysis conducted in this study challenges that perspective by demonstrating that the risky game use group showed no significant differences from the maladaptive game use group, and, in some variables, exhibited even more negative outcomes. Furthermore, males were predominant in all groups except for the general game use group. The higher proportion of males engaging in adaptive game use suggests that men may use gaming adaptively as a means for stress management or social bonding. Conversely, females showed a higher proportion in the general game use group, indicating that gaming for women may primarily serve as leisure or limited activity. However, recent trends indicate that gender differences in gaming motivations are diminishing [2]. This highlights the need for further exploration of the psychosocial factors associated with these observations.

Previous studies using cluster analysis on college and graduate students often failed to distinguish between maladaptive and risky game use groups by combining them into a single cluster [38]. These findings suggest that possessing both adaptive and maladaptive game use does not mitigate the risks associated with maladaptive game use. In fact, some individuals in the risky game use group may appear to engage in adaptive game use. However, it is important to note that they reported experiencing more stress than the maladaptive game use group. A recent report on adaptive game use among gamers over the past year found that the highest-rated factor of adaptive gaming was “emotion and stress management” [2]. Nevertheless, in this study, the risky game use group did not score particularly low on factors related to emotional and stress management. This highlights the complexity of the group and underscores the need for careful attention to the risks associated with game use, even when adaptive elements are present.

Furthermore, only 65.4% of individuals in the risky game use group reported having good family relationships, a notably lower proportion than in the maladaptive game use group (82.3%). In the adaptive game use group, 75.4% of the participants reported high levels of self-control, whereas only 65.1% of the risky game use group did. This finding aligns with previous research, indicating that groups exhibiting both adaptive and maladaptive game use often lack self-control [38]. Additionally, 80.8% of the risky game use group did not acknowledge the concept of online gaming addiction, making them the most resistant to recognizing gaming addiction among the four groups.

In Korea, gaming has become a common leisure activity and a means of stress relief [2], with PC cafes and the spread of smartphones fostering a cultural environment where games serve as a medium for social connection and bonding. As a result, adults in Korea use games not only for personal stress relief but also as a social activity, which may explain the association between adaptive game use and higher subjective well-being found in this study.

On the other hand, due to high academic expectations and strict educational demands in Korean society, there are considerable concerns about gaming overuse, especially among youth, leading to government regulations and a generally negative perception of gaming. These social norms extend into adulthood, contributing to the high levels of stress and lower family relationship quality reported by maladaptive and risky game use groups. Therefore, future research and policies should consider strategies to maintain the positive aspects of gaming while minimizing negative outcomes.

Considering the characteristics of the risky game use group identified in this study, it is evident that this group may exhibit even higher maladaptive game use than the maladaptive game use group. The severity of the risky game use group lies in the fact that the presence of adaptive game use makes it difficult to curb maladaptive game use. These characteristics increase the likelihood of negative responses to family concerns about game use, which may explain why this group reported the highest rate of poor family relationships. Thus, although the risky game use group showed some adaptive game use, it also engaged in maladaptive game use, making it a key target for psychological interventions. This suggests that future policy and program development aimed at addressing gaming overuse should prioritize finding solutions for risky game use groups that exhibit both adaptive and maladaptive use.

It is also important to note that maladaptive game use among adults was not significantly correlated with subjective well-being. This finding aligns with previous research that reported that high-risk groups for Internet gaming addiction among Korean male high school students did not show statistically significant differences in subjective well-being compared to general groups [39]. These results highlight the need for caution, as engaging in extensive game use may not enhance life satisfaction. While the adaptive game use group had significantly higher subjective well-being than the maladaptive game use group, the risky game use group, which exhibited both adaptive and maladaptive game use, showed no significant differences. This highlights the need for further research on this topic. These findings indicate that gaming may not be a suitable coping mechanism when stress increases or happiness decreases, emphasizing the need to explore alternative strategies to improve well-being.

The parenting styles experienced during their developmental years continue to exert a powerful influence on individuals in adulthood. Previous research has shown that parenting styles during childhood significantly affect gaming overuse during adolescence [40], with reports indicating that maladaptive game use in adolescents is influenced by parental behavior [41]. These findings underscore the critical role parents play as a child’s first social relationship in shaping future behaviors. In this study, a correspondence analysis of parenting styles and game use types revealed that democratic parenting styles were closely aligned with healthy game use types. Groups that received appropriate care and affection from their parents, along with discipline when problems arose, were located in close proximity to general or adaptive game use groups. This parenting style extends into adulthood and helps explain adaptive game use. In contrast, risky game use was closely associated with neglectful parenting styles. Neglectful parenting, characterized by a lack of affection and control, is one of the most detrimental styles, and its close relationship with risky game use deserves scrutiny. A study in the U.S. on children’s video gaming and hyperactivity found no significant issues when controlling for parental style, further highlighting the importance of parenting during childhood [42]. However, most prior research has focused on adolescents, with very few studies examining the relationship between parental style and game use in adults. This study is significant as it explores the connection between parenting styles and game use in an adult population.

Regarding psychological variables, the adaptive game use group reported the lowest levels of experiences of depressed mood, although there were no significant differences in anxious mood. Previous studies have shown a significant positive correlation between depressed mood, anxious mood, and online gaming addiction among adolescents [43], with some studies reporting that anxiety, rather than depression, has a more significant impact on gaming and Internet addiction [44,45]. However, research on adults analyzing the effects of mental health on gaming addiction found that the gaming addiction group showed statistically significant differences only in depression, whereas anxiety showed no significant differences, which aligns with the findings of this study [46]. This suggests that for adults, maladaptive game use may be more closely related to depressed mood than to anxious mood. Nonetheless, given the variability in measurement tools for mental health factors such as depressed mood and anxious mood and the need to distinguish between state and trait depression, future research should continue to focus on these issues.

The findings of this study can be better understood through the application of the Flow Theory and the Self-Determination Theory, shedding light on the relationship between game use types and well-being. According to the Flow Theory, when users experience a high state of immersion, they can achieve positive psychological effects through enhanced focus and satisfaction [17]. The observation that the adaptive game use group exhibited higher levels of subjective well-being aligns with this theory, suggesting that these individuals use gaming as a means for stress relief and emotional regulation.

In contrast, the Self-Determination Theory emphasizes the impact of intrinsic and extrinsic motivations on behavior [19]. The high well-being observed in the adaptive game use group appears to stem from the fulfillment of intrinsic motivations such as autonomy, competence, and relatedness. However, the risky game use group, characterized by the coexistence of adaptive and maladaptive game use, tends to be driven by extrinsic motivations. Their reported low levels of self-control and high stress reflect negative psychological outcomes associated with a lack of autonomy. These findings imply that adaptive game use may not necessarily mitigate the negative impacts of maladaptive game use and underscore the importance of intrinsic motivation when analyzing its effects on well-being.

The limitations of this study are as follows. First, because the study focused on individuals in their 20s and 30s in South Korea, the generalizability of the findings to other races and age groups is limited. While most research on gaming addiction has been conducted among adolescents, it is meaningful to study this particular age group as they grew up using PCs and smartphones. Second, psychosocial characteristics were measured using single items rather than validated scales with established reliability and validity, which necessitates caution in interpretation. Although this study explored psychosocial variables, future studies should employ a more precise design. Third, this study did not encompass concurrent analyses of psychopathologies related to gaming or various neurotic traits, and specific contextual factors, such as work-related stress among adults in their 30s, were not thoroughly examined. Future research is encouraged to explore perceived stress across different demographics—such as students and working professionals—in relation to game use to provide a more nuanced understanding of these associations. Fourth, another limitation is the lack of independent consideration for gaming motivations. While this study effectively categorized game use types, it lacks exploration into the underlying motivations for each type, which is crucial for understanding psychological outcomes. For example, motivations such as escapism or stress relief could explain the higher levels of stress observed in maladaptive and risky game use groups, while social or enjoyment-based motivations may account for positive outcomes in the adaptive group [47]. Future research should include these gaming motivations as independent variables to gain a deeper understanding of psychological characteristics. Despite these limitations, this study is significant in that it analyzed the differences in stress and well-being according to game use types among adults in their 20s and 30s and identified significant differences in parenting styles and psychological variables.

## 5. Conclusions

This study examined differences in perceived stress, subjective well-being, and psychosocial variables based on game use type among adult game users. Both the maladaptive and risky game use groups perceived significantly higher levels of stress compared to the general and adaptive game use groups, while the adaptive game use group reported higher subjective well-being than the maladaptive game use group. It was expected that the risky game use group, characterized by both highly adaptive and maladaptive game use, would experience less stress because of their adaptive game use. However, no significant difference in stress perception was found between the risky and maladaptive game use groups. Furthermore, there was no significant difference in subjective well-being, contrary to the expectation that the risky game use group would be healthier than the maladaptive game use group. These findings highlight the need for further social attention to the risky game use group, which may not be as healthy as initially anticipated.

## Figures and Tables

**Table 1:** Descriptive statistics and correlation coefficients of the study variables (*n* = 300).

Variables	Maladaptive Game Use	Perceived Stress	Subjective Well-Being	M ± SD
Adaptive game use	0.37 ***	0.06	0.13 *	34.53 ± 10.78
Maladaptive game use	1.00	0.30 ***	−0.04	14.09 ± 5.56
Perceived stress		1.00	−0.57 ***	28.25 ± 4.99
Subjective well-being			1.00	45.64 ± 11.54

* *p* < 0.05, *** *p* < 0.001.

**Table 2:** Differences in self-regulation and school adjustment by game use group (*n* = 300).

Game Use Type	Adaptive Game Use		Maladaptive Game Use	
	M ± SD	F (*p*)	M ± SD	F (*p*)
General game use ^(a)^	23.04 ± 6.66	186.35 (<0.001)	10.55 ± 2.00	419.98 (<0.001)
Adaptive game use ^(b)^	40.96 ± 6.16	a < c < b < d *	11.48 ± 1.73	a,b < c < d *
Maladaptive game use ^(c)^	35.03 ± 6.22		19.47 ± 2.93	
Risky game use ^(d)^	48.46 ± 6.54		26.00 ± 4.52	

^abcd^ Alphabets represent each group. * Results from Scheffé’s post hoc test.

**Table 3:** Differences in perceived stress and subjective well-being by game use type (*n* = 300).

Game Use Type	Perceived Stress		Subjective Well-Being	
	M ± SD	F (*p*)	M ± SD	F (*p*)
General game use ^(a)^	27.72 ± 4.94	11.33 (<0.001)	45.39 ± 12.13	3.62 (0.014)
Adaptive game use ^(b)^	26.85 ± 4.63	a,b < c,d *	47.34 ± 11.28	b > c *
Maladaptive game use ^(c)^	30.44 ± 4.84		41.82 ± 11.12	
Risky game use ^(d)^	31.15 ± 4.20		48.19 ± 9.40	

^abcd^ Alphabets represent each group. * Results from Scheffé’s post hoc test.

**Table 4:** Differences in game use type by demographic variables (*n* = 300).

Variables	Categories	Game Use Group				χ^2^ (*p*)
		General	Adaptive	Maladaptive	Risky	
		*n* (%)	*n* (%)	*n* (%)	*n* (%)	
Gender	Male	30 (19.0)	76 (48.1)	35 (22.2)	17 (10.8)	30.13
	Female	68 (47.9)	38 (26.8)	27 (19.0)	9 (6.3)	(<0.001)
Age group	20′	46 (30.7)	58 (38.7)	32 (21.3)	14 (9.3)	0.62
	30′	52 (34.7)	56 (37.3)	30 (20.0)	12 (8.0)	(0.892)
Marital status *	Marriage	31 (41.3)	29 (38.7)	11 (14.7)	4 (5.3)	4.60
	Single	67 (30.5)	84 (38.2)	48 (21.8)	21 (9.5)	(0.204)
Satisfaction with economic level	Low	71 (34.6)	69 (33.7)	48 (23.4)	17 (8.3)	6.45
	High	27 (28.4)	45 (47.4)	14 (14.7)	9 (9.5)	(0.092)
Perceived physical health	Healthy	29 (31.2)	35 (37.6)	22 (23.7)	7 (7.5)	0.88
	Unhealthy	69 (33.3)	79 (38.2)	40 (19.3)	19 (9.2)	(0.830)
Perceived mental health	Healthy	28 (30.8)	31 (34.1)	20 (22.0)	12 (13.2)	3.86
	Unhealthy	70 (33.5)	83 (39.7)	42 (20.1)	14 (6.7)	(0.277)

* For marital status, we analyzed data from 295 respondents, excluding widowed and other respondents (5).

**Table 5:** Differences in psychosocial characteristics by game use type (*n* = 300).

Variables	Categories	Game Use Group				χ^2^ (*p*)
		General	Adaptive	Maladaptive	Risky	
		*n* (%)	*n* (%)	*n* (%)	*n* (%)	
Awareness of online game addiction	No	37 (37.8)	90 (78.9)	41 (66.1)	21 (80.8)	43.01
	Yes	61 (62.2)	24 (21.1)	21 (33.9)	5 (19.2)	(<0.001)
Self-control	Low	22 (22.4)	17 (14.9)	15 (24.2)	6 (23.1)	10.96
	High	76 (77.6)	97 (85.1)	47 (75.8)	20 (76.9)	(0.012)
Experiences of depressed mood	Low	67 (68.4)	86 (75.4)	34 (54.8)	14 (53.8)	9.37
	High	31 (31.6)	28 (24.6)	28 (45.2)	12 (46.2)	(0.019)
Experiences of anxious mood	Low	43 (43.9)	64 (56.1)	30 (48.4)	12 (46.2)	3.39
	High	55 (56.1)	50 (43.9)	32 (51.6)	14 (53.8)	(0.335)
Relationships with family	Bad	15 (15.3)	7 (6.1)	11 (17.7)	9 (34.6)	15.89
	Good	83 (84.7)	107 (93.9)	51 (82.3)	17 (65.4)	(0.001)
Communication between family members	Bad	32 (32.7)	28 (24.6)	23 (37.1)	11 (42.3)	4.92
	Good	66 (67.3)	86 (75.4)	39 (62.9)	15 (57.7)	(0.178)

**Table 6:** Differences in psychosocial characteristics by game use type (*n* = 300).

Variables	Categories	Game Use Group				*p*
		General	Adaptive	Maladaptive	Risky	
		*n* (%)	*n* (%)	*n* (%)	*n* (%)	
Perceived parenting style	Democratic	75 (34.2)	91 (41.6)	40 (18.3)	13 (5.9)	0.014 *
	Permissive	7 (25.9)	8 (29.6)	10 (37.0)	2 (7.4)	
	Authoritarian	11 (33.3)	10 (30.3)	8 (24.2)	4 (12.1)	
	Neglectful	5 (23.8)	5 (23.8)	4 (19.0)	7 (33.3)	

* Fisher–Freeman–Halton exact test.

## Data Availability

The data presented in this study are not available elsewhere.
